# Progressive Colonization of Bacteria and Degradation of Rice Straw in the Rumen by Illumina Sequencing

**DOI:** 10.3389/fmicb.2017.02165

**Published:** 2017-11-06

**Authors:** Yanfen Cheng, Ying Wang, Yuanfei Li, Yipeng Zhang, Tianyi Liu, Yu Wang, Thomas J. Sharpton, Weiyun Zhu

**Affiliations:** ^1^Jiangsu Key Laboratory of Gastrointestinal Nutrition and Animal Health, Laboratory of Gastrointestinal Microbiology, Nanjing Agricultural University, National Center for International Research on Animal Gut Nutrition, Nanjing, China; ^2^Departments of Microbiology and Statistics, Oregon State University, Corvallis, OR, United States

**Keywords:** rice straw, fiber degradation, ruminal bacteria, carbohydrate-active enzymes, metagenome

## Abstract

The aim of this study was to improve the utilization of rice straw as forage in ruminants by investigating the degradation pattern of rice straw in the dairy cow rumen. Ground up rice straw was incubated *in situ* in the rumens of three Holstein cows over a period of 72 h. The rumen fluid at 0 h and the rice straw at 0.5, 1, 2, 4, 6, 12, 24, 48, and 72 h were collected for analysis of the bacterial community and the degradation of the rice straw. The bacterial community and the carbohydrate-active enzymes in the rumen fluid were analyzed by metagenomics. The diversity of bacteria loosely and tightly attached to the rice straw was investigated by scanning electron microscopy and Miseq sequencing of 16S rRNA genes. The predominant genus in the rumen fluid was *Prevotella*, followed by *Bacteroides*, *Butyrivibrio*, unclassified *Desulfobulbaceae*, *Desulfovibrio*, and unclassified *Sphingobacteriaceae*. The main enzymes were members of the glycosyl hydrolase family, divided into four categories (cellulases, hemicellulases, debranching enzymes, and oligosaccharide-degrading enzymes), with oligosaccharide-degrading enzymes being the most abundant. No significant degradation of rice straw was observed between 0.5 and 6 h, whereas the rice straw was rapidly degraded between 6 and 24 h. The degradation then gradually slowed between 24 and 72 h. A high proportion of unclassified bacteria were attached to the rice straw and that *Prevotella*, *Ruminococcus*, and *Butyrivibrio* were the predominant classified genera in the loosely and tightly attached fractions. The composition of the loosely attached bacterial community remained consistent throughout the incubation, whereas a significant shift in composition was observed in the tightly attached bacterial community after 6 h of incubation. This shift resulted in a significant reduction in numbers of *Bacteroidetes* and a significant increase in numbers of *Firmicutes*. In conclusion, the degradation pattern of rice straw in the dairy cow rumen indicates a strong contribution by tightly attached bacteria, especially after 6 h incubation, but most of these bacteria were not taxonomically characterized. Thus, these bacteria should be further identified and subjected to functional analysis to improve the utilization of crop residues in ruminants.

## Introduction

The global demand for animal-source foods is estimated to undergo a dramatic increase by 2030, due to the predicted growth in the human population (FAO, 2011). One challenge facing increased animal production in China is the limited availability of high-quality forage such as alfalfa (for example, China needed to import approximately 0.9 million tons of alfalfa in 2014^1^). By contrast, China generates large annual amounts of cellulosic crop residues, such as rice straw and wheat straw, with 0.7 billion tons of crop residues produced in 2009 alone (Cai et al., 2011). Elimination of these crop residues by combustion pollutes the air and results in smog, as well as being a waste of energy. Therefore, the use of these residues as a livestock feed for ruminants could represent a beneficial and more environmentally friendly disposal route for this cellulosic material.

Weimer et al. (2009) described the rumen as the most elegant and highly evolved cellulose-digesting system in nature. The microbiota in the rumen is responsible for the degradation of cellulose by colonization of ingested roughage and excretion of fiber-degrading enzymes. The colonization of ingested roughage has been investigated in several publications. Edwards et al. (2007), who investigated the colonization of perennial ryegrass by rumen bacteria, found colonization by a diverse and consistent population of ruminal bacteria, whose numbers increased rapidly within 5 min of roughage incubation. Huws et al. (2013, 2016), who examined degradation of perennial ryegrass in the cow rumen by denaturing gradient gel electrophoresis and 16S rRNA gene sequencing, revealed the development of a distinct primary bacterial community (*Butyrivibrio*, *Prevotella*, *Selenomonas*, and *Fibrobacter*) within 2 h of incubation and a secondary bacterial community (*Butyrivibrio*, *Fibrobacter*, *Pseudobutyrivibrio*, and *Selenomonas*) after 4 h of incubation. Piao et al. (2014) reported two major shifts in bacterial community composition during *in situ* incubation of switchgrass: The first shift was the colonization of *Bacteroidia* and *Clostridia* within 30 min and the second was an enrichment of *Spirochaete* and *Fibrobacteria* classes after 16 h of incubation. Liu et al. (2016) demonstrated a significant shift after 6 h incubation of both rice straw and alfalfa and significant difference in the bacterial community between the two forages. Koike et al. (2014), who investigated the colonization patterns of bacteria between untreated rice straw and sodium hydroxide-treated rice straw, found that the population sizes of fibrolytic species were significantly higher in sodium hydroxide-treated rice straw. Wang et al. (2016), who investigated the shifts in rumen fermentation and microbial community fed with different types of carbohydrates, found that rice straw increased acetate and hydrogen production and decreased the ratio of acetate propionate. For the excretion of fiber-degrading enzymes, Patel et al. (2014) reported that the carbohydrate-active enzymes (CAZymes) in the buffalo rumen included carbohydrate binding modules (CBM), carbohydrate esterases (CE), glycosyl hydrolases (GH), glycosyl transferases (GT), and polysaccharide lyases (PL), with the dominant enzymes being those of the GH family, including cellulases, hemicellulases, oligosaccharide-degrading enzyme, and debranching enzymes. Brulc et al. (2009) demonstrated that GH2 and GH3 were predominant in the rumen of bovine. The authors also suggested that the initial colonization of fiber was by microbes possessing enzymes, which attacked the easily available side chains of complex plant polysaccharides. Hess et al. (2011) investigated the biomass-degrading genes and genomes in the rumen of cow by metagenomic analysis and demonstrated most of identified sequences were belonging to endoglucanase, glucosidase, and cellobiohydrolase.

The aim of the present study was to investigate the degradation pattern of rice straw by ruminal bacteria and to identify the bacteria that colonize rice straw during its digestion in the cow rumen. A better understanding of ruminal digestion of rice crop residues will have dual benefits for China, as diversion of these residues to roughage feed for ruminants will improve animal husbandry while reducing air pollution from combustion.

## Materials and Methods

### Sample Collection

The rice straw was dried at 65°C, ground into ∼0.5 mm pieces, and weighed into individual nylon bags (2.5 g/bag). A total of 54 bags were prepared and placed into the rumens of three cannulated Holstein cows (18 bags per cow). The cow diet consisted of 15% corn, 2% wheat bran, 10% soybean meal, 0.7% calcium carbonate, 0.8% calcium orthophosphate, 0.5% sodium chloride, 1% premix, 20% corn silage, 30% Chinese wildrye, and 20% alfalfa hay [on a dry matter (DM) basis]. The premix contained, per kilogram: Fe 5, 500 mg, Cu 4, 080 mg, Zn 17, 500 mg, Mn 4, 980 mg, Se 110 mg, I 180 mg, Co 88.5 mg, VA >2,000,000 IU, VD_3_ 600,000 IU, and VE 10,800 mg. The animals were fed three times per day, at 4:00 a.m., 10:30 a.m., and 4:00 p.m. All animal procedures were carried out under the protocols approved by the Animal Care and Use Committee of Nanjing Agricultural University, 1999.

Two nylon bags were retrieved from each cow’s rumen at 0.5, 1, 2, 4, 6, 12, 24, 48, and 72 h (a total of six bags for each time point) and washed gently with phosphate-buffered saline (PBS) (pH 7.4) to remove the rumen contents on the outer surface of the bag. A few residue fragments were removed from each bag and fixed with 2.5% glutaraldehyde for scanning electron microscopy (SEM). Samples (1 g) of the residues were then used to isolate the associated fraction (As) from the adherent fraction (Ad), as described previously (Larue et al., 2005). These fractions were stored at -20°C until analysis of the loosely and tightly attached bacteria. The remaining residues were washed and dried at 65°C for the analysis of relative degradation of rice straw biomass. At 0, 24, 48, and 72 h, 50 ml samples of rumen fluid were collected from each cow for the analysis of bacterial community and CAZyme profiles.

### Fiber Degradation Analysis

Relative biomass degradation of rice straw was determined by DM, crude fiber (CF), neutral detergent fiber (NDF), and acid detergent fiber (ADF) analysis. DM and CF were analyzed according to AOAC (2000). NDF and ADF were determined by the method of Van Soest et al. (1991).

### Bacterial Community and CAZyme Analysis of Rumen Fluid by Metagenome Shotgun Sequencing

The rumen fluid samples collected from all time points for each cow were mixed and used for DNA extraction. A 10 ml volume of each mixed rumen fluid was centrifuged and the resulting pellet was ground in liquid nitrogen for DNA extraction with CTAB buffer. The DNA was purified in an equal volume of phenol/chloroform/isoamyl alcohol (25:24:1). The final DNA pellets were washed with ice cold 70% ethanol and dissolved in TE buffer.

The metagenomic DNA libraries from the three cows were prepared following the manufacturer’s instructions (Illumina), and the samples were then pair-end sequenced using Illumina Hiseq4000 at BGI (Shenzhen, China). A total of 119,215,228 reads with a length of 150 bp was obtained after removing the reads containing N and the adapter (SRA accession number: SRP094501). The forward sequencing reads from each sample were quality controlled with meta-qc^2^ and the cow genome (NCBI assembly: GCA_000003205.4) was used as reference to remove the reads derived from the host. A total of 19,447,862, 19,311,185, and 19,452,969 sequences remained after quality control for each cow. MetaPhlAn (Segata et al., 2012) was then used to analyze the bacterial composition and ShotMAP (Nayfach et al., 2015) was used to annotate with the CAZy database^3^.

### Analysis of Bacteria Loosely and Tightly Attached to Rice Straw by Illumina MiSeq Sequencing of 16S rRNA Gene

The samples from the Ad (0.25 g) and As (1 ml) fractions were used for DNA extraction using the bead-beating and phenol–chloroform extraction method described by Zoetendal et al. (1998). The universal primers (341F 5′-CCTAYGGGRBGCASCAG-3′ and 806R 5′-GGACTACNNGGGTATCTAAT-3′) (Behrendt et al., 2012) were used to obtain the PCR amplicons for paired-end sequencing on an Illumina MiSeq platform at Novogene (Beijing, China). The paired-end reads (SRA accession number: SRP066537) were assembled, trimmed, quality-filtered, and analyzed according to Li et al. (2016). Briefly, the paired-end reads were first assembled and trimmed with PANDAseq (Bartram et al., 2011) to remove primers and barcodes, as well as sequences less than 400 bp, longer than 500 bp, or with ambiguous bases. The trimmed sequences were then filtered by ea-utils (Aronesty, 2011) to remove sequences with mean quality score values less than 30. Subsequently, the quality-filtered sequences were analyzed by QIIME for open reference OTU picking, alpha and beta diversity analysis at a sequence depth of 17,900 sequences, and taxonomic assignment based on Greengenes (Caporaso et al., 2010; McDonald et al., 2012). The functional prediction and differential characterization were conducted with PICRUSt and LEfSe (Segata et al., 2011; Langille et al., 2013).

### Scanning Electron Microscopy

After fixation for more than 2 h, the samples were washed three times with PBS (pH 7.4), prior to sequential dehydration using 50, 70, 80, 90, and 100% ethanol. The ethanol was then replaced with tertiary butyl alcohol before critical point drying using a Hitachi freeze dryer (ES-2030, Hitachi, Tokyo, Japan). The dried samples were then sputter coated with approximately 10 nm of Au/Pd using a Hitachi E-1010 Ion sputter instrument (Hitachi, Tokyo, Japan). SEM imaging was performed on a Hitachi S-3000 N microscope (Hitachi, Tokyo, Japan).

### Statistical Analysis

Data were presented as mean ± SEM (*n* = 6). The statistical analysis was carried out using General Linear Model procedure with SPSS 22 (SPSS Inc., Chicago, IL, United States). Duncan test was used to compare the means. Statistical significance was defined at *P* < 0.05.

## Results

### Bacterial Community and CAZyme Profiles in the Rumen Fluid by Metagenome Shotgun Sequencing

**FIGURE 1:**
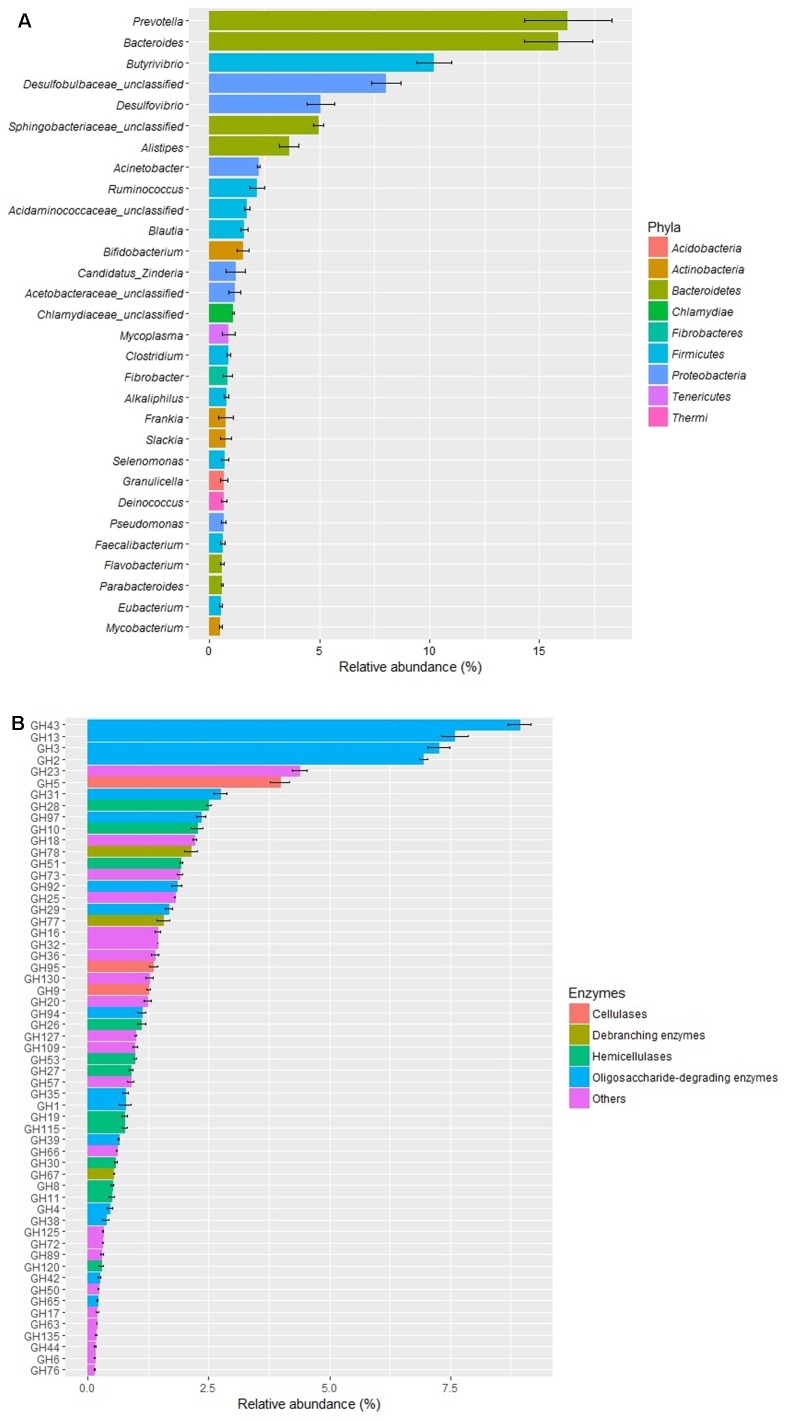
Relative abundances of predominant genera (>0.5% relative abundance, **A**) and GH families **(B)** in the rumen fluid of cows by metagenome shotgun sequencing (*n* = 3).

The metagenomic analysis of the rumen fluid showed that the relative abundances of *Prevotella* (16.3%), *Bacteroides* (15.9%), and *Butyrivibrio* (10.2%) were above 10%, followed by unclassified *Desulfobulbaceae* (8.0%), *Desulfovibrio* (5.1%), and unclassified *Sphingobacteriaceae* (5.0%) (**Figure 1A**). The analysis of CAZymes profiles showed that all the five enzyme classes (GHs, GTs, PLs, CEs, and AAs) were detected in the rumen fluid (data not shown). The diversity profile of the GH families (**Figure 1B**) showed that GH43 was the most abundant family, with a relative abundance of 8.9%, followed by GH13 (7.6%), GH3 (7.3%), and GH2 (6.9%). Among the four categories of cellulases, hemicellulases, debranching enzymes, and oligosaccharide degrading enzymes, the oligosaccharide degrading enzymes from the 16 GH families were the most abundant (43.8%), followed by hemicellulases (13.0%), cellulases (6.6%), and debranching enzymes (4.2%).

### Biomass Degradation of Rice Straw in the Rumen

Approximately 13% of the DM was degraded within 0.5 h (Supplementary Figure 1). And then, no significant change of DM digestibility was observed between 0.5 and 6 h. Subsequently, the rice straw was rapidly degraded between 6 and 24 h with ∼17% of DM being degraded. After 24 h of incubation, the degradation of rice straw gradually slowed down with ∼10% of DM being degraded between 24 and 48 h and ∼7% of DM being degraded between 48 and 72 h. The digestibility of CF, NDF, and ADF was similar to the digestibility of DM (data not shown). The SEM photos (Supplementary Figure 2) showed the dynamics of the degradation of rice straw over the 72 h incubation period in the rumen.

### Temporal Changes in the Community of Bacteria Loosely and Tightly Attached to Rice Straw by Illumina MiSeq Sequencing of 16S rRNA Gene

The temporal digestibility profiles of DM, CF, NDF, and ADF were used as a basis for selecting samples collected at 0.5, 6, 24, and 72 h for bacterial community analysis. A total of 1,946,473 sequences remained from 48 samples after quality-filtering with a mean of 40,551 ± 11,108 sequences per sample. A total of 4820 OTUs were identified at the 97% similarity level.

Alpha diversity analysis of loosely and tightly attached bacteria (**Table 1**) showed that the PD_whole_tree, Chao1, observed species, and Shannon and Simpson indexes were significantly different between the two fractions (*P* < 0.05). The incubation time significantly (*P* < 0.05) affected the PD_whole_tree, observed species, and Shannon index, while the interaction between fraction and time significantly (*P* < 0.05) affected all of the aforementioned metrics.

**Table 1:** Alpha diversity of bacteria loosely and tightly attached to rice straw after incubation for 0.5, 6, 24, and 72 h in the rumen of cows.

Alpha diversity^#^	Loosely attached				Tightly attached				SEM	*P*-values		
			
	0.5	6	24	72	0.5	6	24	72		Fraction	Time	Fraction ^∗^ time
Good’s coverage	0.940	0.936	0.937	0.935	0.938	0.939	0.938	0.942	0.0004	0.008	0.432	0.001
PD_whole_tree	303.0	317.2	307.2	317.2	292.3	293.9	293.0	284.2	1.8	<0.001	0.005	<0.001
Chao1	3987	4184	4104	4215	4069	4001	3986	3791	24.5	<0.001	0.345	<0.001
Observed species	2956	3141	3049	3164	2964	2951	2884	2773	19.9	<0.001	0.009	<0.001
Shannon index	9.94	10.12	10.10	10.22	10.04	10.01	9.78	9.61	0.03	<0.001	0.021	<0.001
Simpson	0.996	0.997	0.997	0.998	0.997	0.997	0.996	0.995	0.0002	0.049	0.223	<0.001

^#^*Calculating at a depth of 17,900 sequences*.

**FIGURE 2:**
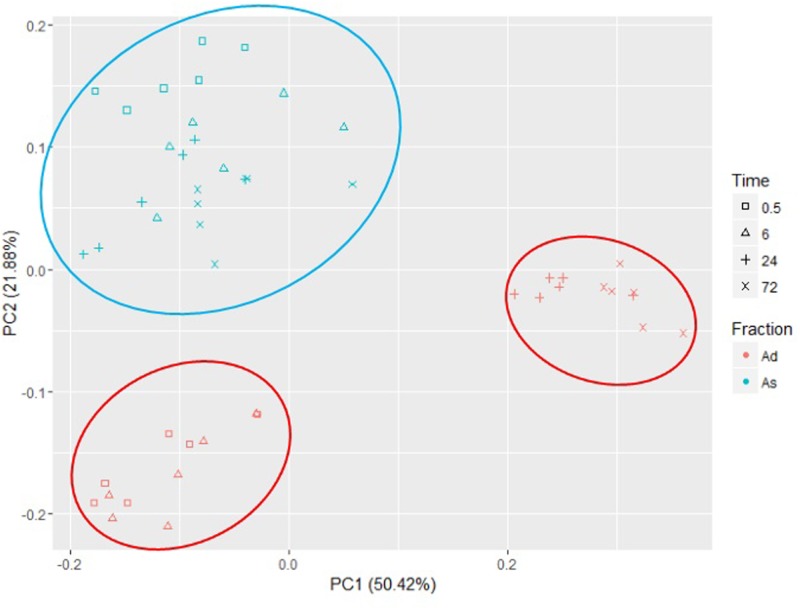
Principal component analysis of bacteria loosely (As) and tightly (Ad) attached to rice straw after 0.5, 6, 24, and 72 h incubation in the rumen of cows by Illumina MiSeq sequencing of 16S rRNA gene (*n* = 6).

Taxonomic assignment of the OTUs showed that Bacteroidetes and Firmicutes were the dominant phyla in all samples, accounting for ∼92% and ∼97% in the loosely and tightly attached fractions, respectively (Supplementary Figure 3). Principal component analysis of the bacteria attached to rice straw (**Figure 2**) showed that all the samples from the loosely attached fraction clustered together in a separate group. By contrast, the samples from the tightly attached fraction separated into two groups according to the incubation time.

**FIGURE 3:**
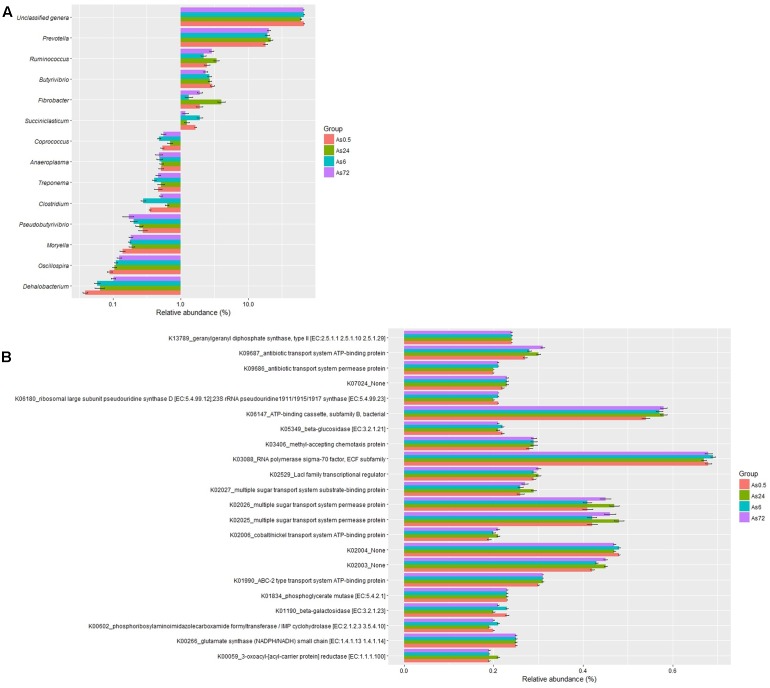
Relative abundances **(A)** and predicted function **(B)** of bacteria loosely (As) attached to rice straw after 0.5, 6, 24, and 72 h incubation in the rumen of cows by Illumina MiSeq sequencing of 16S rRNA gene. (*n* = 6; the relative abundances of bacteria above 0.1% or the relative abundances of KEGG Orthologies above 0.2% in at least one sample were presented.)

The relative abundances of 36 genera were above 0.1% in at least one sample in the loosely attached fraction. A total of 23 genera were unidentified and their accumulative relative abundances were 67.0, 66.7, 60.3, and 65.4% at 0.5, 6, 24, and 72 h, respectively. The remaining 13 genera were classified as *Prevotella*, *Fibrobacter*, *Butyrivibrio*, *Ruminococcus*, *Succiniclasticum*, *Clostridium*, *Dehalobacterium*, *Coprococcus*, *Moryella*, *Pseudobutyrivibrio*, *Oscillospira*, *Treponema*, and *Anaeroplasma*. *Prevotella* was the predominant classified genus, accounting for 18.1, 19.2, 21.7, and 20.4% at 0.5, 6, 24, and 72 h, respectively. None of the other classified genera accounted for more than 3% in the samples (**Figure 3A**). The predicted function of the loosely attached bacteria (**Figure 3B**) showed a diversity of fiber-degrading genes.

**FIGURE 4:**
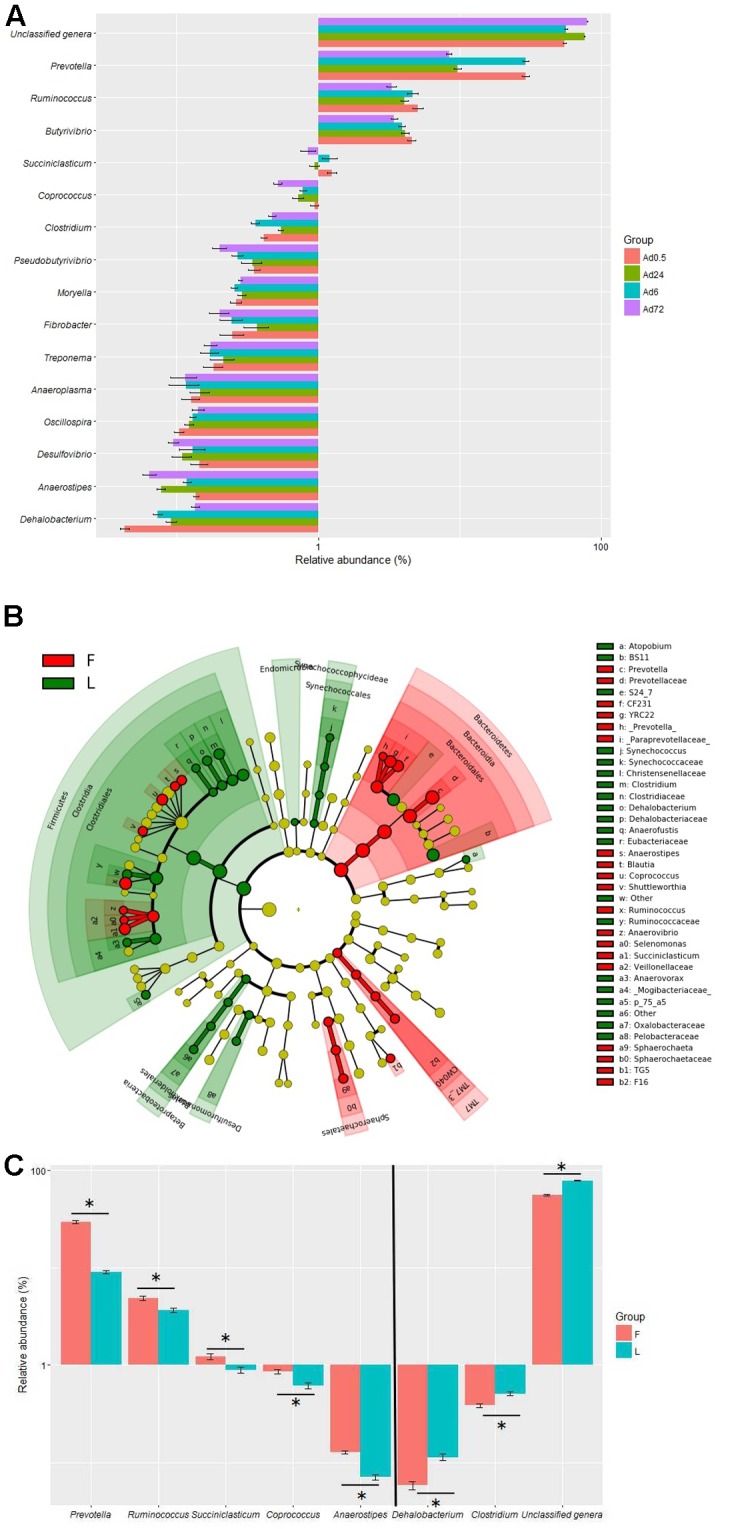
Relative abundances **(A)** of bacteria tightly (Ad) attached to rice straw after 0.5, 6, 24, and 72 h incubation in the rumen of cows by Illumina MiSeq sequencing of 16S rRNA gene (the relative abundances of bacteria above 0.1% in at least one sample were presented) and LEfSe analyses **(B)** of the bacterial community shift. **(C)** The significant (*P* < 0.05) shift genera. (*n* = 6; F, the bacteria community at 0.5 and 6 h; L, the bacteria community at 24 and 72 h.)

The relative abundances of 36 genera were also above 0.1% in at least one sample in the tightly attached fraction. A total of 21 genera were unclassified and their accumulative relative abundances were 54.6, 56.2, 75.8, and 79.3% at 0.5, 6, 24, and 72 h, respectively. The remaining 15 genera were classified as *Prevotella*, *Fibrobacter*, *Butyrivibrio, Ruminococcus*, *Succiniclasticum*, *Clostridium*, *Dehalobacterium*, *Coprococcus*, *Moryella*, *Pseudobutyrivibrio*, *Oscillospira*, *Treponema*, *Anaeroplasma*, *Desulfovibrio*, and *Anaerostipes* (**Figure 4A**). The predicted function of the tightly attached bacteria also showed a diversity of fiber-degrading genes (data not shown). LEfSe analysis of the microbial shift showed that *Bacteroidetes*, *Sphaerochaetales*, and TM7 clusters were significantly higher at 0.5 and 6 h, while *Firmicutes*, *Betaproteobacteria*, *Synechococcophycideae*, and *Desulfuromonadales* were significantly higher at 24 and 72 h (**Figure 4B**). At the genus level, the proportions of *Prevotella*, *Ruminococcus*, *Succiniclasticum*, *Coprococcus*, and *Anaerostipes* significantly decreased, while the proportions of *Dehalobacterium*, *Clostridium*, and unclassified genera were significantly increased at 24 and 72 h (**Figure 4C**).

## Discussion

Rice is the predominant dietary energy source in China, and its production was approximately 204 million tons in 2013 (National Bureau of Statistics of China, 2014). According to Kadam et al. (2000), about 1.35 tons of crop residues remain in the field for every ton of crop harvested, so more than 250 million tons of rice straw were produced in China in 2013. The use of rice crop residues to supplement the short supply of high-quality forages in China is therefore an attractive proposition. However, studies on the degradation of these crop residues in ruminants are still needed.

Previous work has included the study by Chen et al. (2017), who investigated the effects of *Lactobacillus plantarum* on the *in vitro* degradation of maize and rice straws. Similarly, Xin et al. (2015) investigated the effects of different treatments on the *in vitro* degradation of rice straw, whereas Sun et al. (2015) investigated the improvement of *in vitro* degradation of rice straw by silkworm excrement. Koike et al. (2014) investigated the improvement of the degradation of rice straw by sodium hydroxide treatment. Polyorach and Wanapat (2015) investigated the improvement of the rice straw quality by the addition of urea and calcium hydroxide. Liu et al. (2016) investigated the degradation pattern of rice straw and alfalfa in the rumen. Wang et al. (2016) investigated the rumen fermentation and microbial community of lactating dairy cows fed with rice straw and corn silage. However, only limited studies considered the dependence of rice straw degradation on the colonization of rice straw and excretion of fiber-degrading enzymes by the rumen microbial community.

The metagenomic analysis in the present study showed that oligosaccharide-degrading enzymes (especially GH2, GH3, GH13, and GH43) predominate in the rumen fluid, which might result from an enrichment in oligosaccharides arising from degradation of plant fiber by cellulases, debranching enzymes, and hemicellulases. Brulc et al. (2009) investigated the fiber-degrading enzymes of the fiber-adherent microbes in the rumen of bovine and found that oligosaccharide-degrading enzymes, especially GH2 and GH3, were predominant. Hess et al. (2011) investigated the biomass-degrading genes and genomes from cow rumen and also found that oligosaccharide-degrading enzymes, especially endoglucanase, glucosidase, and cellobiohydrolase, were predominant. Patel et al. (2014) conducted a metagenomics study of the microbial and CAZyme profiles of buffalo rumen and found that oligosaccharide-degrading enzymes, especially GH2 and GH3, were predominant. Similarly, the metagenomic analysis by Pitta et al. (2016) also revealed that oligosaccharide-degrading enzymes, especially GH3 and GH13, predominated in the rumen contents. Dai et al. (2015) showed that GH2 and GH3 were predominant by metagenomics as well, but metatranscriptomic analysis indicated that the relative percentage of oligosaccharide-degrading enzymes was dramatically reduced, indicating that the expression of these enzymes, and perhaps other CAZymes as well, might be induced by their substrates. Many more studies are needed to elucidate the mechanism underlying the expression of these CAZymes for improvement of the degradation of fibers in the rumen.

The dominant bacterial phyla in both loosely and tightly attached fractions were *Bacteroidetes* and *Firmicutes.* An association between bacteria belonging to the two phyla and the degradation of fiber and polysaccharides has been previously reported (Wright and Klieve, 2011; Zened et al., 2012). However, Lee et al. (2009) reported that *Firmicutes* often carried 4–15 rRNA operons, which implied that the 16S rRNA sequencing might lead to an overestimation of the abundance of *Firmicutes*.

In the present study, the predominant classified genus found attached to rice straw was *Prevotella*, followed by *Ruminococcus* and *Butyrivibrio*. *Prevotella* predominates not only in the rumen (Edwards et al., 2004; Kong et al., 2010; Mao et al., 2015), but also in the foregut of kangaroos (Li et al., 2016) and in the gut of African children (De Filippo et al., 2010). This genus was reported to have moderately saccharolytic potential (Shah and Collins, 1990) and can metabolize various sugars (including xylan) to acetate, propionate, and succinate (Sakamoto and Ohkuma, 2012). Ueki et al. (2007) isolated a novel *Prevotella* species from plant residue and rice roots and demonstrated that this strain had the ability to degrade xylan and hemicellulose. *Ruminococcus* is another well-studied rumen microorganism with high fiber-degrading ability (Latham and Wolin, 1977; Christopherson et al., 2014; Rozman et al., 2015). A recent study by Dai et al. (2015) indicated that *Ruminococcus* produced the largest proportion of cellulases in the rumen, as well as large amounts of hemicellulases and oligosaccharide-degrading enzymes. Another common rumen microorganism is *Butyrivibrio*, first reported by Bryant and Small (1956) as an anaerobic, butyric acid-producing and curved rods genus. Mao et al. (2015) reported that *Butyrivibrio* accounted for ∼3% in the rumen digesta and ∼12% in the mucosa of dairy cattle. Dai et al. (2015) reported that *Butyrivibrio* could produce ∼7% of the oligosaccharide-degrading enzymes and a limited amount of hemicellulases in the cow rumen. Liu et al. (2016) investigated the temporal dynamics of ruminal bacterial microbiota colonizing rice straw within ruminants and found that *Prevotella*, *Ruminococcus*, *Butyrivibrio*, *Fibrobacter*, and *Treponema* were predominant (>1% in at least one sample). Interestingly, the authors reported that the relative abundance of *Fibrobacter* was significantly increased after 6 h incubation (up to 5.29% at 16 h), which was not observed in the present study. Similarly, the authors reported high amount of *Treponema* after 6 h incubation (4.15% at 48 h), which was remained ∼0.5% over the incubation.

However, in the present study, we found that a high proportion of unclassified genera were involved in the degradation of rice straw. The relative abundance of unclassified genera, which were tightly attached to the rice straw, was ∼80% after 72 h incubation. Piao et al. (2014) also reported a relative abundance of ∼62% for unclassified genera tightly attached to switchgrass after 72 h incubation in the rumen. These findings imply that these unclassified genera might be very important in the degradation of plant fiber; therefore, the function of these microorganisms needs further investigation.

Our PCoA indicated that the group of loosely attached bacteria remained relatively stable during the 72 h incubation. However, the tightly attached bacterial community changed after 6 h of incubation. Huws et al. (2013, 2016), who investigated the successional colonization of perennial ryegrass by the bacteria in the cow rumen, identified distinct primary (0–2 h) and secondary (4 h onward) bacterial communities. These authors also demonstrated that a proportion of the primary colonizing bacteria (*Succinivibrio* spp.) detached after 2 h of incubation and were replaced with the population of secondary colonizing bacteria, which included *Prevotella*, *Pseudobutyrivibrio*, *Roseburia*, and *Ruminococcus*. In the present study, we found that the proportions of *Anaerostipes*, *Coprococcus*, *Desulfovibrio*, *Prevotella*, *Pseudobutyribivrio*, *Ruminococcus*, and *Succiniclasticum* all significantly decreased in the secondary bacterial community when compared to the primary bacterial community. For instance, the *Prevotella* significantly decreased to ∼9% abundance in the secondary bacterial community from ∼29% in the primary bacterial community, whereas the proportions of *Clostridium*, *Dehalobacterium*, and *Oscillospira* increased in the secondary bacterial community. Liu et al. (2016), who investigated the temporal dynamics of rumen bacteria colonizing rice straw and alfalfa hay, also identified distinct primary (0–6 h) and secondary (6 h onward) bacterial communities. Members of *Spirochaetes* and *Fibrobacteria* were predominant after 6 h incubation in the rumen. Piao et al. (2014) also observed a similar microbial shift when they investigated the temporal dynamics of fibrolytic microorganisms with switchgrass as substrate, again implying that the primary and secondary bacterial communities might have different functions in the degradation of plant fiber. However, the roles of these bacterial communities and their relationship to the chemical components of plant fiber require further investigation.

## Conclusion

The present study documented the degradation pattern of rice straw within the cow rumen. Within 30 min of incubation, the rice straw was colonized by bacteria that included *Prevotella*, *Ruminococcus*, *Butyrivibrio*, and a large proportion of unclassified bacteria. After 6 h of incubation, a significant shift in the population of tightly attached bacteria was observed, resulting in decreases in *Anaerostipes*, *Blautia*, *Butyrivibrio*, *Coprococcus*, *Desulfovibrio*, *Prevotella*, *Pseudobutyrivibrio*, *Ruminococcus*, and *Succiniclasticum* and increases in *Clostridium*, *Dehalobacterium*, and *Oscillospira*. Most of the bacteria involved in the degradation of rice straw were unidentified; thus, further investigation of the function of these bacteria, and especially the secondary bacterial community, is needed to improve the utilization of crop residues in ruminants.

## Author Contributions

Conceived and designed the experiments: YC and WZ. Performed the experiments: YiW, YL, YZ, TL, and YuW. Generated and analyzed the data: YiW, YL, YZ, TL, YuW, YC, and TS. Wrote the paper: YC, TS, and WZ. All authors read and approved the final manuscript.

## Conflict of Interest Statement

The authors declare that the research was conducted in the absence of any commercial or financial relationships that could be construed as a potential conflict of interest.

## Footnotes

1 www.customs.gov.cn/

2 https://github.com/sharpton/meta-qc

3 www.cazy.org
