# Supplementary material for: Progressive Colonization of Bacteria and Degradation of Rice Straw in the Rumen by Illumina Sequencing
Source: Front Microbiol. 2017 Nov 6;8:2165. doi: 10.3389/fmicb.2017.02165 (PMC5681530; doi:10.3389/fmicb.2017.02165)
Supplement: Supplementary file 1 [file Data_Sheet_1.docx]

*Supplementary Material*

**Progressive colonization of bacteria and degradation of rice straw in the rumen by Illumina sequencing**

**Yanfen Cheng^1^, Ying Wang^1^, Yuanfei Li^1^, Yipeng Zhang^1^, Tianyi Liu^1^, Yu Wang^1^, Thomas J Sharpton^2^, and Weiyun Zhu^1*^**

**Correspondence**: Weiyun Zhu: [zhuweiyun@njau.edu.cn](mailto:zhuweiyun@njau.edu.cn)


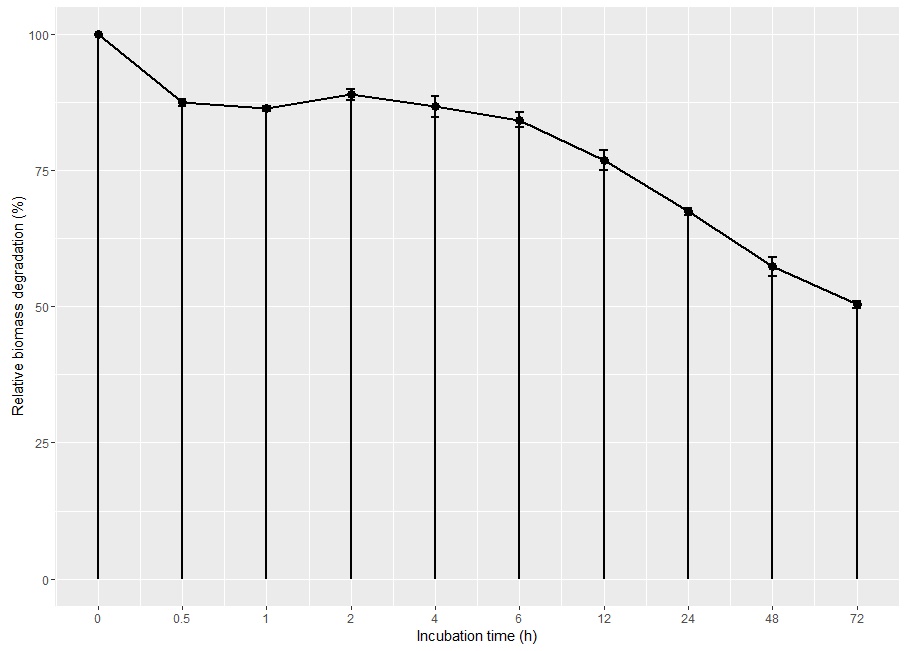


**Supplementary Figure 1.** Dry matter degradation of rice straw over 72 h incubation in the rumen of cows (n=6).


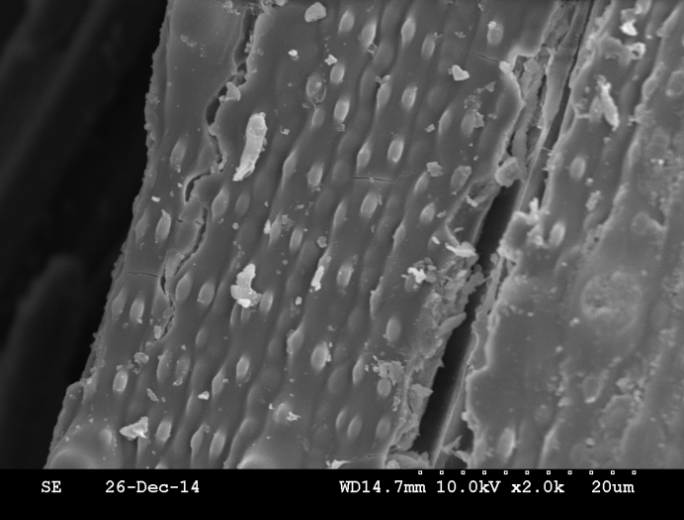

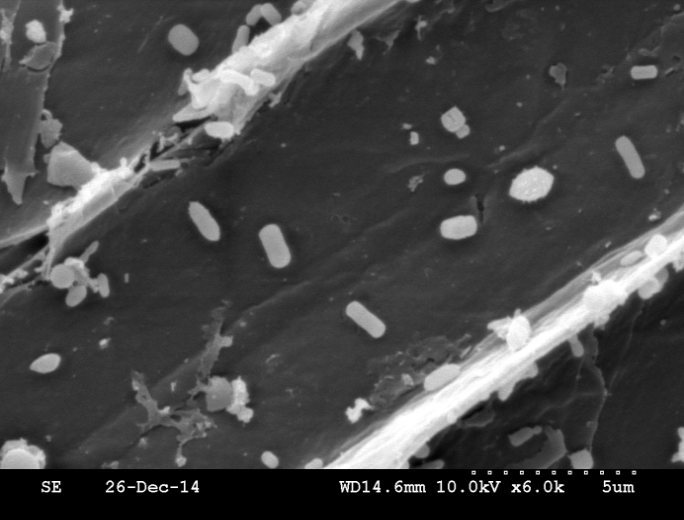

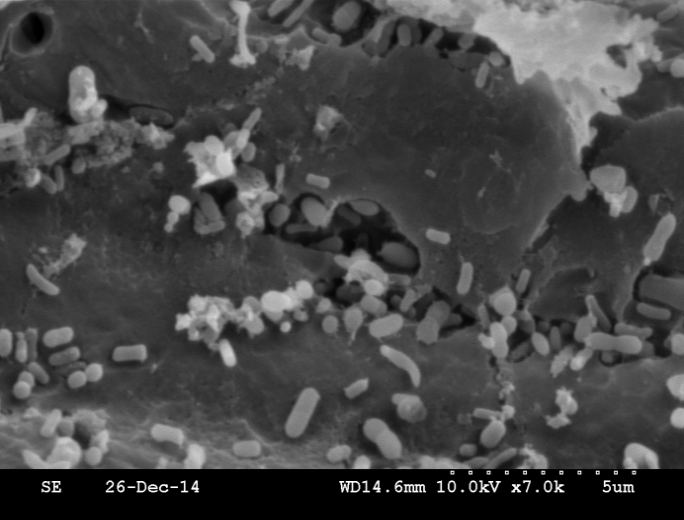


C

B

A


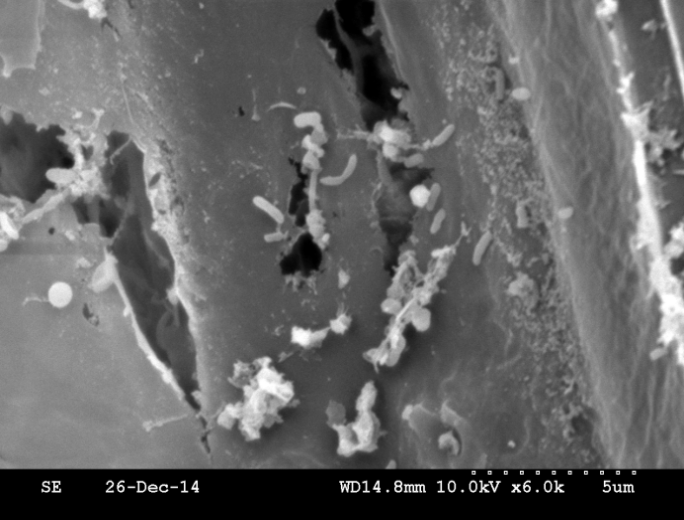

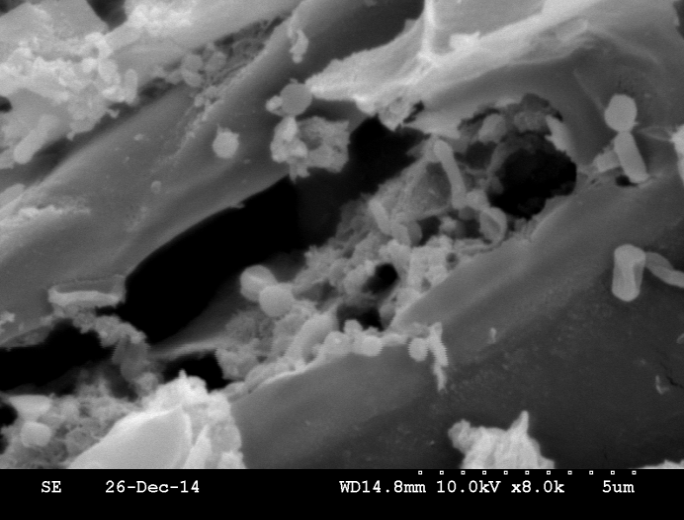


E

D

**Supplementary Figure 2.** Scanning electron microscopy photos showing the degradation of rice straw by attached microorganisms before (A) and after incubation for 0.5 (B), 6 (C), 24 (D) and 72 h (E) in the rumen of dairy cow. Scale bars were 5μm except A which was 20 μm.


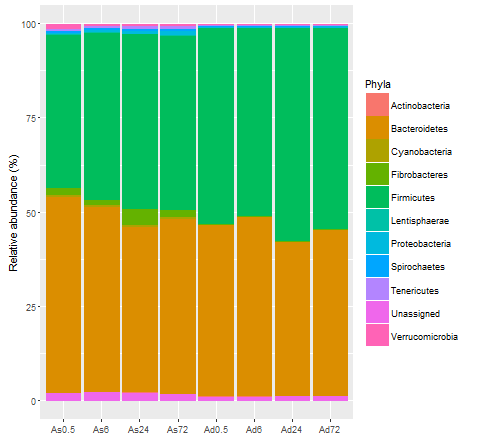


**Supplementary Figure 3.** Relative abundances of bacterial phyla (> 0.1% in at least one sample) loosely (As) and tightly (Ad) attached to rice straw after 0.5, 6, 24 and 72 h incubation in the rumen of cows.
